# Distinct HIV-1 entry phenotypes are associated with transmission, subtype specificity, and resistance to broadly neutralizing antibodies

**DOI:** 10.1186/1742-4690-11-48

**Published:** 2014-06-23

**Authors:** Kelechi Chikere, Nicholas E Webb, Tom Chou, Katharina Borm, Jasminka Sterjovski, Paul R Gorry, Benhur Lee

**Affiliations:** 1 Department of Microbiology, Immunology, and Molecular Genetics, Los Angeles, USA; 2 Department of Biomathematics, University of California at Los Angeles, Los Angeles, CA, USA; 3 Center for Biomedical Research, Burnet Institute, Melbourne, VIC, Australia; 4 Department of Infectious Diseases, Monash University, Melbourne, VIC, Australia; 5 Department of Microbiology and Immunology, University of Melbourne, Melbourne, VIC, Australia; 6 Department of Microbiology, Icahn School of Medicine at Mount Sinai, One Gustave L. Levy Place, #1124, New York, NY 10029, USA

**Keywords:** Virus entry, CD4, CCR5, Receptor usage efficiency, Acute transmitted/founder envelopes, Subtype C, Broadly neutralizing antibodies, Receptor affinity profiling

## Abstract

**Background:**

The efficiency of CD4/CCR5 mediated HIV-1 entry has important implications for pathogenesis and transmission. The HIV-1 receptor affinity profiling (Affinofile) system analyzes and quantifies the infectivity of HIV-1 envelopes (Envs) across a spectrum of CD4/CCR5 expression levels and distills these data into a set of Affinofile metrics. The Affinofile system has shed light on how differential CD4/CCR5 usage efficiencies contributes to an array of Env phenotypes associated with cellular tropism, viral pathogenesis, and CCR5 inhibitor resistance. To facilitate more rapid, convenient, and robust analysis of HIV-1 entry phenotypes, we engineered a reporter Affinofile system containing a Tat- and Rev-dependent *Gaussia* luciferase-e*G*FP-*R*eporter (GGR) that is compatible with the use of pseudotyped or replication competent viruses with or without a virally encoded reporter gene. This GGR Affinofile system enabled a higher throughput characterization of CD4/CCR5 usage efficiencies associated with differential Env phenotypes.

**Results:**

We first validated our GGR Affinofile system on isogenic JR-CSF Env mutants that differ in their affinity for CD4 and/or CCR5. We established that their GGR Affinofile metrics reflected their differential entry phenotypes on primary PBMCs and CD4+ T-cell subsets. We then applied GGR Affinofile profiling to reveal distinct entry phenotypes associated with transmission, subtype specificity, and resistance to broadly neutralizing antibodies (BNAbs). First, we profiled a panel of reference subtype B transmitted/founder (T/F) and chronic Envs (n = 12) by analyzing the infectivity of each Env across 25 distinct combinations of CD4/CCR5 expression levels. Affinofile metrics revealed that at low CCR5 levels, our panel of subtype B T/F Envs was more dependent on high levels of CD4 for HIV-1 entry compared to chronic Envs. Next, we analyzed a reference panel of 28 acute/early subtype A-D Envs, and noted that subtype C Envs could be distinguished from the other subtypes based on their infectivity profiles and relevant Affinofile metrics. Lastly, mutations known to confer resistance to VRC01 or PG6/PG19 BNAbs, when engineered into subtypes A-D Envs, resulted in significantly decreased CD4/CCR5 usage efficiency.

**Conclusions:**

GGR Affinofile profiling reveals pathophysiological phenotypes associated with varying HIV-1 entry efficiencies, and highlight the fitness costs associated with resistance to some broadly neutralizing antibodies.

## Background

Human immunodeficiency virus type 1 (HIV-1) enters target cells through the stepwise interaction of its envelope glycoproteins (Env) with CD4 and a coreceptor, either CCR5 or CXCR4. Receptor binding induces a series of conformational changes that results in fusion pore formation and virus/cell membrane fusion [1]. Acutely transmitted viruses invariably use CCR5 (R5) regardless of the subtype. Furthermore, although CXCR4-using (X4, R5X4) viruses can emerge in approximately 40-50% of late stage HIV-1 subtype B infections [2,3], most HIV-1 infected subjects, particularly those with subtype A and C viruses [4-6], progress to late stages of infection despite exclusively harboring R5 viruses.

While many viral and host factors contribute to HIV-1 progression, there is a strong body of evidence that supports some Env determinants of pathogenicity. For example, in patients with R5 viruses, isolates from late stages of infection have a greater capacity to infect macrophages [7-9], which correlates with more efficient usage of the low levels of CD4 and CCR5 expressed on these cells [9-13]. These late stage R5 isolates can also cause increased levels of cell-cell fusion [14] and CD4+ T-cell apoptosis [15]. Late stage brain isolates have also been shown to utilize low levels of CD4 and/or CCR5 for entry [16-24]. Therefore, viruses capable of exploiting limiting levels of CD4 and/or CCR5 may have expanded target cell tropism with pathological consequences [24-26]. Furthermore, viruses that are resistant to the CCR5 antagonists vicriviroc (VVC) and maraviroc (MVC) exhibit a reduced ability to use lower levels of CCR5 compared to their non-resistant counterparts [27-29]. Finally, the recent characterization of transmitter/founder (T/F) Envs has indicated that these R5 variants enter and replicate in activated primary T-cells but not macrophages [30], underscoring the increasingly evident notion that CCR5 usage is not equivalent to macrophage-tropism [5,31]. Together, these studies show that the efficiency with which a viral Env engages CD4 and/or CCR5 can have an influence on pathogenicity, disease progression and resistance to CCR5 antagonists [5,32,33]. Therefore, a more refined understanding of how Env-CD4/CCR5 usage develops and differs under alternate evolutionary histories will inform the development and use of HIV-1 vaccines and therapeutics that target HIV-1 entry.

The Affinofile system, based on a CD4 and CCR5 dual-inducible cell line, permits quantitative characterization of HIV-1 infection across 24–48 distinct combinations of CD4/CCR5 expression levels [34]. Multiple groups have used this receptor affinity profiling system (Affinofile) to reveal unique CD4/CCR5 usage efficiencies associated with distinct pathophysiological phenotypes. These studies have shed light on the nature of CCR5-inhibitor resistance [27-29,35-37], the relationship between CD4/CCR5 usage and cellular tropism as well as disease pathogenesis [38], and CD4/CCR5 usage interdependence (reviewed in [39]). Using this system, the infectivity of an Env under 24–48 distinct combinations of CD4 and CCR5 expression is compiled and summarized as three metrics that collectively describe a distinct profile of CD4 and CCR5 usage. Biological insights are gained by comparing the Affinofile metrics of different Envs. Affinofile metrics can be extracted from infectivity data by an automated web-based computational platform [34].

Comprehensive infectivity profiling requires the examination of each Env under multiple distinct combinations of CD4/CCR5 expression levels. To gain further insights into HIV-1 entry phenotypes associated with distinct pathophysiologies, and to examine a larger panel of Envs from distinct cohorts, we engineered a higher throughput, second generation Affinofile system that would: (1) improve the robustness of the infectivity data obtained, (2) ease the process of data sampling and analysis by permitting sequential time-point sampling of the infected cell supernatant without the need for end-point lysis, and (3) allow infectivity measurements without requiring a virus-associated reporter gene while retaining compatibility with any HIV-1 proviral backbone used for Env pseudo typing. To this end, we transduced Affinofile cells with a Tat- and Rev-dependent reporter engineered to express green fluorescent protein (GFP) and secrete *Gaussia* luciferase into the supernatant upon infection. This Gaussia luciferase-GFP reporter (GGR) Affinofile cell line now permits simple and rapid detection of HIV-1 infection by serial sampling a small volume of supernatant for Gaussia luciferase activity, while also taking full advantage of the CD4 and CCR5 inducibility of the original Affinofile cells.

In this study, we validate our new GGR Affinofile system, and use this improved, higher throughput GGR Affinofile system to reveal distinct Env phenotypes associated with acute transmission, subtype specificity and neutralization resistance.

## Results

### Generation and characterization of the GGR Affinofile cell line

**Figure 1:**
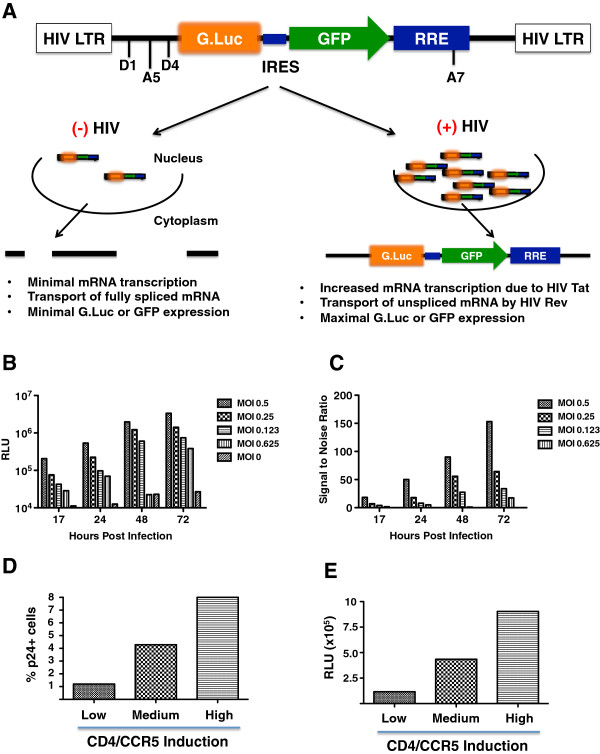
**Generation and characterization of the GGR Affinofile Cell Line. (A)** Schema of the tat-rev dependent *Gaussia* luciferase (gLuc)-IRES-GFP reporter vector as described in the text. **(B)** and **(C)** GGR cells were maximally induced with doxycyline (Doxy, 4ng/ml) and ponasterone A (PonA, 4 μM) at the time of their seeding in 96-well plates. 16–21 hours post-seeding/induction, cells were infected with wt JR-CSF virus at varying multiplicities of infection (MOI). The titer of the virus was previously determined on stable CD4/CCR5-expressing GHOST cells where CD4/CCR5 levels are non-limiting. At 17, 24, 48, and 72 hpi, 10 μL (out of 150) of the infected cell supernatant was removed and analyzed for gLuc activity as per manufacturer’s instructions. Luciferase activity (measured as relative light units, RLU), and the corresponding signal:noise ratios at each data point are shown in **(B)** and **(C)**, respectively. Mock-infected cell supernatant served as the background signal. **(D)** and **(E)** GGR cells were induced at high (3.2ng/mL Doxy, 2 μM PonA), medium (1.6ng/mL Doxy, 1μm PonA), and low (0.4ng/mL Doxy, 0.25μM PonA) levels, and infected as above with pseudotyped virus at an MOI of 0.25. Three days post-infection, supernatant was collected and analyzed for gluc expression **(E)**, while cells from each well were individually processed for intracellular p24 staining **(D)** as described in methods. Data shown is representative of two independent experiments.

We modified a previously published Tat/Rev-dependent vector [40,41] by cloning the *Gaussia* luciferase (GLuc) gene upstream of an eGFP reporter gene, linked via an internal ribosomal entry site (IRES) (Figure 1A). Judiciously placed splice donor and acceptor sites, in addition to the Rev-responsive element (RRE) placed downstream of the eGFP reporter gene, ensures that only the full-length, unspliced reporter mRNA will be translated in the presence of Tat and Rev, which is provided by commonly used HIV-1 reporter vectors and replication-competent HIV-1. Lentiviral VSV-G pseudotypes containing this *G*Luc-e*G*FP *R*eporter (GGR) vector were used to transduce early passage Affinofile cells. Stable *GGR* Affinofile cell lines with optimal properties were single cell cloned as described in methods.To determine the ability of GGR Affinofile cells to detect HIV-1 infection, we infected a stable clone of GGR Affinofile cells (at maximum CD4/CCR5 induction) using a range of viral inoculums (JR-CSF, MOI = 0.5 – 0.0625) and serially sampled the infected cell culture supernatant for GLuc activity. GLuc activity could be detected at 20-fold above background as early as 17 hpi depending on the amount of viral inoculum used (Figure 1B-C). Furthermore, we observed that GLuc activity in the infected culture supernatant mirrored the level of infection as reported by intracellular p24 staining (Figure 1D-E), especially at low MOIs (e.g. 0.2) that ensure a single infectious event per cell.

### Defining the parameters that impact the infectivity metrics used for profiling HIV-1 entry efficiency

**Figure 2:**
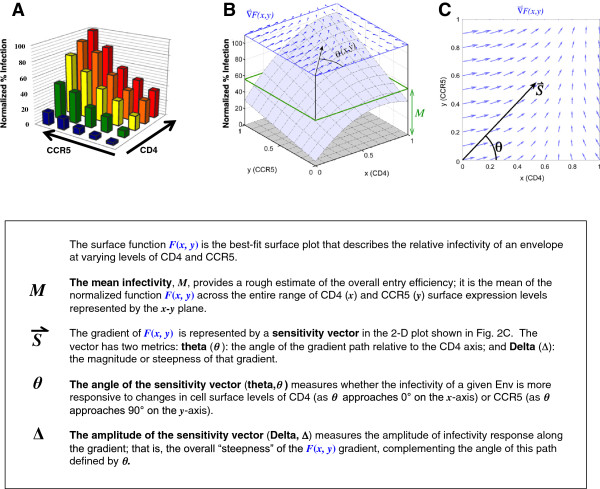
**Defining the limiting parameters of sensitivity vector metrics used for profiling HIV-1 entry efficiency. (A)** Infectivity of a primary subtype B R5-virus monitored across 25 distinct combinations of CD4 and CCR5 expression levels. The normalized infectivity profile is shown as a 3-D bar graph with the luciferase activity obtained at the highest CD4 and CCR5 induction level set at 100%. **(B-C)** The surface function *F*(*x*, *y*) is used to fit the infectivity data as previously described [34]. The resulting 3-D surface plot can be represented by three metrics that reflect distinct phenotypic properties of the infecting virus envelope: **(B)** the mean infectivity level (*M*)*,* and **(C)** the angle (*θ*) and amplitude (Δ) of the sensitivity vector $\overset{⇀}{S}$ that describes the envelope’s response to varying levels of CD4 and CCR5. For clarity, the operational definitions of these metrics, and what they measure with respect to the infectious phenotype of Env, are also indicated. Note that while we have changed the nomenclature of these Affinofile metrics to more intuitively reflect the Env properties they are intended to describe, the fundamental definitions are the same as in Johnston et al. (Ref [34]). Thus, “mean induction” is now termed “mean infectivity”, and vector “magnitude” is now termed vector “amplitude”.

We previously demonstrated that R5 virus infection of Affinofile cells across a spectrum of CD4 and CCR5 expression levels generated an infectivity profile (Figure 2A) that can be fitted by the surface function *F*(*x*, *y*) to give the surface plot shown in Figure 2B. *F*(*x*, *y*) describes the infectivity response as a function of CD4 and CCR5 cell surface expression levels [34]. The salient features of this surface function can be captured by three biophysically meaningful parameters illustrated in Figure 2B and C: the mean infectivity level *M* (Figure 2B), and the angle and amplitude of the sensitivity vector ( $\overset{⇀}{S}$) representing the gradient of the surface function *F*(*x*, *y*) on a 2-D plot (Figure 2C). Mean infectivity (*M*) expresses the overall infectivity observed across all levels of CD4 and CCR5 expression. The gradient of *F*(*x*, *y*) is fit by the sensitivity vector ( $\overset{⇀}{S}$) shown in Figure 2C, representing both the stoichiometric combination of CD4 and CCR5 with the greatest impact on entry across the entire surface (*θ*) and the magnitude of that impact (∆) illustrated by the vector field in Figure 2C. For example, a relative increase in *θ*, driven by a shift in the gradient toward the CCR5 axis (Figure 2C), indicates a greater responsiveness to CCR5. The magnitude of this shifted responsiveness may be comparatively larger (increased ∆) or smaller (decreased ∆), indicating a relative increase in CCR5 usage efficiency or a decrease in both CD4 and CCR5 usage efficiency, respectively. The operational definitions of these parameters are indicated in the panels below Figure 2A-C. Their mathematical definitions and formulations have been reviewed recently [39]. Together, these three metrics quantitatively describe the phenotypic behavior of a given viral envelope in response to changes across a spectrum of CD4 and CCR5 expression levels.

Similar to regular Affinofile cells, GGR Affinofile cells can be used to characterize a range of distinct Env phenotypes (see Additional file 1: Figure S1A-C) and the infectivity profile of each Env can be represented by the set of three metrics (Additional file 1: Figure S1D-F). Notably, all three metrics (*θ, ∆, M*) for a given Env can be represented on a polar plot and are highly reproducible under standardized conditions (Additional file 1: Figure S1G).

### Affinofile metrics illuminate the phenotype of functionally well-characterized point mutants

**Figure 3:**
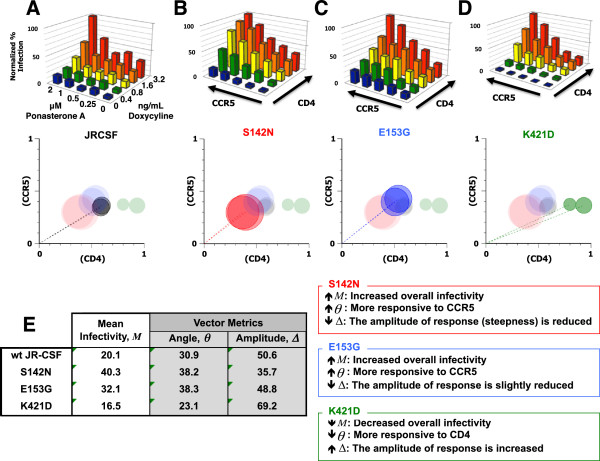
**Sensitivity vector metrics further illuminate the phenotype of well-characterized point mutants. (A)** Infectivity profile of wt JR-CSF (R5) envelope, and three point mutants: **(B)** S142N, **(C)** E153G and **(D)** K421D, previously shown to enhance or perturb CCR5 or CD4 usage with polar plots (beneath) representing the metrics obtained from mathematical analysis of the infectivity profiles **(A-C)**. The vector angle (*θ*) is the angle between the x-axis and the dotted line. The vector amplitude (*Δ*) is represented by the length of the dotted line. The mean infectivity (*M*) is represented by the size of the circle. Data shown is a representative of two experiments. **(E)** Table of the average Affinofile metrics obtained from **(A-D)** and graphically shown in polar plots beneath **(A-D)**. Boxes next to **(E)** describe the phenotypes indicated by each metric relative to wt JR-CSF. The infectivity profile of each Env was independently repeated twice.

To further define the biological meaning of the three Affinofile metrics, we examined three point mutants in JR-CSF with well-described effects on CD4 and CCR5 binding. S142N [42] and E153G [43] are both V1 loop mutations that increase the ability of JR-CSF to enter cells with low levels of CCR5 [20,25] or CD4, respectively, while K421D is a “bridging sheet” mutant that reduces the affinity of gp120 for CCR5 [44,45]. Viruses pseudotyped with wild type (wt) JR-CSF, or with S142N or K421D Env mutants were produced and titrated first on Ghost-R5 cells. An equivalent MOI (0.2) of each pseudotype was then used to infect GGR Affinofile cells expressing 25 distinct combinations of CD4 and CCR5 levels. We are cognizant that viral titers are cell-type dependent, but we reasoned that normalizing the infectious inoculum on GGR Affinofile cells using titers obtained from infecting Ghost-R5 cells (where CD4/CCR5 levels are non-limiting) would fairly reveal biologically relevant differences in entry efficiencies when CD4/CCR5 levels do become limiting under certain induction conditions on GGR Affinofile cells.Compared to wt JR-CSF (Figure 3A), the S142N mutant exhibited enhanced entry at every level of CCR5 at or above a specific threshold level of CD4 (0.4 ng/ml Doxy) (Figure 3B, compare the rows of green, yellow, orange and red bars along the CCR5 axis with Figure 3A). A similar increase in infection was observed for E153G, particularly at low CD4 expression (compare blue and green bars in Figure 3C to A), whereas the K421D mutant showed inefficient entry at low CCR5 levels regardless of how much CD4 was present (Figure 3D, note the low infectivity at 0 and 0.25 μM PonA (<20% of maximum) even when CD4 was maximally induced). S142N was more responsive to changes in CCR5 levels than wt JR-CSF, and this phenotype was reflected as an increase in from 30.5° to 38° for wt JR-CSF and S142N, respectively. Recall that a relative increase or decrease in vector angle indicates that an Env’s infectivity is more responsive to changes in levels of CCR5 or CD4, respectively. A summary of the Affinofile metrics is given in Figure 3E, and illustrated in the polar plots below Figures 3A-D.

For S142N, the ability to use CCR5 efficiently also enhanced its infectivity at any given level of CD4; thus, the overall level of infection across the entire matrix of CD4/CCR5 expression is higher. This overall increase in infectivity is reflected in the increase in *M* from 20 to 40.3 for wt JR-CSF and S142N, respectively (Figure 3E, and also graphically represented by the size of the circle in the polar plot below Figure 3B). This combination of an increase in *θ* and *M* support the conclusion that S142N uses CCR5 more efficiently.

E153G, which putatively confers the ability to use low levels of CD4, also exhibited an increased *M* (32.1) compared to wt JR-CSF (20.1), illustrating that these mutations, both attributed to usage of low CD4 or CCR5 expression, have a broad impact on infectivity across all combinations of CD4 and CCR5. This highlights the inter-dependence of CD4 and CCR5 usage as, for example, a higher CD4 binding affinity is likely to increase the success of gp120-CCR5 engagement. E153G exhibited a stronger response to CCR5 expression than CD4 compared to wt JR-CSF, which is reflected in an increased angle (38°, Figure 3E), matching the same responsive phenotype observed for S142N. That E153G would necessarily result in a lower, or more CD4-responsive, angle than wt JR-CSF or S142N is not obvious given the proposed indirect mechanism by which this mutation primes Env to use low levels of CD4. E153G is positioned distal to the CD4 binding site at the apex of the Env trimer and also results in a higher neutralization sensitivity to the V3 loop conformational Mab 447-52D [43]. Our data supports the conclusion of Clapham and colleagues, that the ability to use low levels of CD4 attributed to E153G is not the direct result of CD4 engagement, but the result of a more fluid and successful transition to CCR5 recognition due to the mutation’s effect on V1/V2 mobility [43]. These results extend the phenotype originally ascribed to E153G, visible as an increased infectivity at low levels of CD4 relative to wt JR-CSF (compare blue and green bars in Figure 3C to A), into a more complex interplay of both CD4 and CCR5 that supports the role of this mutation in facilitating CCR5 recognition.

In contrast, K421D exhibited inefficient entry at low levels of CCR5, which is consistent with the known role of this K421 bridging sheet residue in mediating coreceptor interactions [44,45]. Interestingly, at high CCR5 levels (2 and 1 μM PonA), K421D responded more dramatically to increasing levels of CD4 than wt JR-CSF (Figure 3C). These phenotypic properties are reflected by a decrease in θ (30.9° to 23.1° for wt JR-CSF and K421D, respectively), and a concomitant increase in Δ (50.6 to 69.2 for wt and K421D, respectively) (Figure 3D and E). Just as an increase in θ indicates that the S142N and E153G Envs are more responsive to changes in levels of CCR5 expression when compared to wt JR-CSF, a decrease in θ indicates that the K421D Env is more responsive to changes in CD4 levels. The increase in amplitude for K421D is apparent because the differential magnitude of response is markedly greater for K421D at the highest CCR5 and CD4 levels, which is related to the relative lack of infectivity response at low CD4/CCR5 levels. Recall that the amplitude measures the “steepness” of the steepest direction along the surface function *F*(*x,y*) used to fit the infectivity data (Figure 2, box). Overall, the mean infectivity (*M*) for K421D was only moderately decreased compared to wt JR-CSF (16.5 vs 20.1, Figure 3D and E). This likely reflects a balance between the lack of infectivity observed at low CD4/CCR5 levels, and the compensatory increase in the magnitude of K421D’s infectivity response at high CD4/CCR5 levels. These results, collectively, reveal that high levels of CD4/CCR5 may compensate for the inefficient entry exhibited by the K421D mutation at low CCR5 levels. A summary of these metric comparisons and their meaning is included next to Figure 3E.

### Affinofile infectivity profile and metrics reflect biologically relevant differences in T-cell tropism

**Figure 4:**
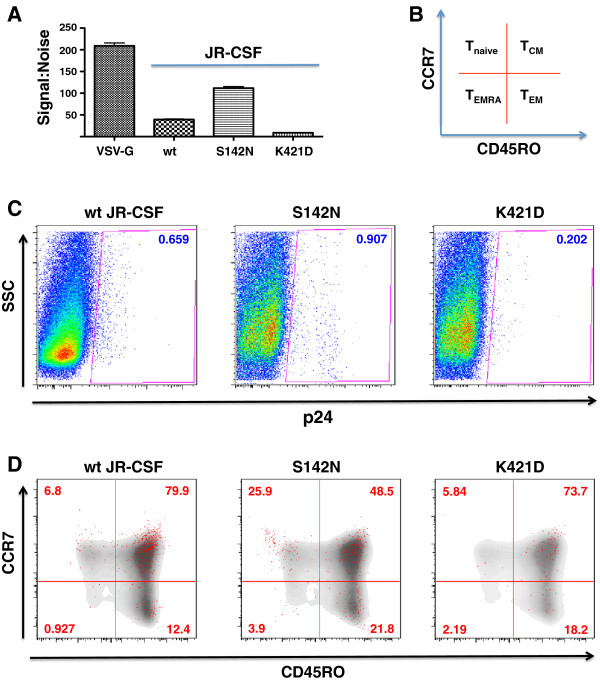
**Sensitivity vector metrics reflect biologically relevant differences in T cell subset tropism. (A)** Total PBMCs were infected with luciferase reporter pseudotypes bearing wt, S142N, or K421D JR-CSF envelopes. VSV-G pseudotypes were used as positive controls. All infections (except for VSV-G) could be inhibited by maraviroc (>95%). Error bars represent ranges between two experiments. **(B)** Scheme for using CCR7 (PE-Cy7) and CD45RO (FITC) to identify the following T-cell subsets: Naïve (CCR7+ CD45RO-), Central Memory (T_CM_, CCR7+ CD45RO+), Effector Memory (T_EM_, CCR7- CD45RO+), and Effector Memory RA (T_EMRA_, CCR7- CD45RO-). **(C)** and **(D)** CD8-depleted PBMCs were infected with the indicated pseudotyped viruses at an MOI of 20 (as titered on Ghost-R5 cells). Three days post-infection, cells were analyzed by multi-color flow cytometry. **(C)** Infected cells were identified by intracellular p24 staining using PE-conjugated KC57 Mab. **(D)** Uninfected T-cell subset distribution is shown in grey density plot, while infected p24+ cells are overlaid as the red dots. The percent of total p24+ cells are indicated in each quadrant. All infections could be inhibited by maraviroc (>90%). Data shown here is a representative of two independent donors.

To determine how these Affinofile metrics reflect the ability of a viral Env to infect primary CD4+ T-cells, we infected total PBMCs with pseudotyped luciferase reporter viruses bearing wt JR-CSF, S142N or the K421D Env mutants. Figure 4A shows that the S142N virus infected PBMCs better than wt JR-CSF while the K421D virus exhibited the lowest level of infection. This pattern reflected the *θ* and *M* metrics of the respective viruses, as the limiting parameter on primary CD4+ T-cells are the levels of CCR5 (low), not CD4 (high).

Next, we infected CD3/CD28 stimulated CD4+ T-cells with wt JR-CSF, S142N or the K421D Env pseudotyped virus, and assessed the infection of the indicated CD4+ T-cell subsets (Figure 4B) via intracellular p24 staining and multiparametric FACS analysis three days post-infection. The overall levels of infection, as determined by the percentage of p24+ cells, were consistent with the luciferase reporter results observed in Figure 4A, with S142N infecting the greatest proportion of cells and K421D the lowest (Figure 4C). In most cases, the majority of p24+ cells were CD4+ T-central memory cells (T_CM_, CCR7 + CD45RO+), with the remainder comprising the effector memory subset (T_EM_, CCR7-CD45RO+) or the naïve T-cell subset (T_naive_, CCR7 + CD45RO-) (Figure 4D). It is unclear whether the small number of p24+ cells found in CD4+ T-effector RA + cells (T_EMRA_, CCR7-CD45RO-) represents a reproducibly infectable population since CD4+ T_EMRA_ cells are thought to be non-permissive for R5 virus infection [46].

Interestingly, the S142N mutant demonstrated not only an increase in overall infectivity, but also an altered pattern of cellular tropism. Compared to wt JR-CSF, the S142N mutant infected almost 4-fold more naïve T-cells (25.9% vs 6.8%) and 2-fold more T_EM_ cells (21.8% vs 12.4%). As a consequence, S142N infected fewer T_CM_ cells compared to wt JR-CSF (48.5% vs 79.9%) (Figure 4D). Although K421D infected fewer CD4+ T-cells, the CD4+ T-cell subset distribution resembled that of wt JR-CSF infection. Thus, the differential ability to use CCR5 as quantified by the GGR Affinofile assay is reflected in the differential ability of the wt and mutant JR-CSF Envs (S142N) to infect CD4+ T-cell subsets where relatively high and uniform CD4 expression is coupled to relatively low and variable CCR5 expression [20,46]. Our results indicate that the distinct entry efficiencies quantified by our GGR Affinofile system reflect the biologically relevant contributions of CD4 and CCR5 usage to primary CD4+ T-cell subset tropism.

### Affinofile metrics reveal differences in CD4/CCR5 usage efficiencies between chronic and transmitter/founder derived Envs

**Figure 5:**
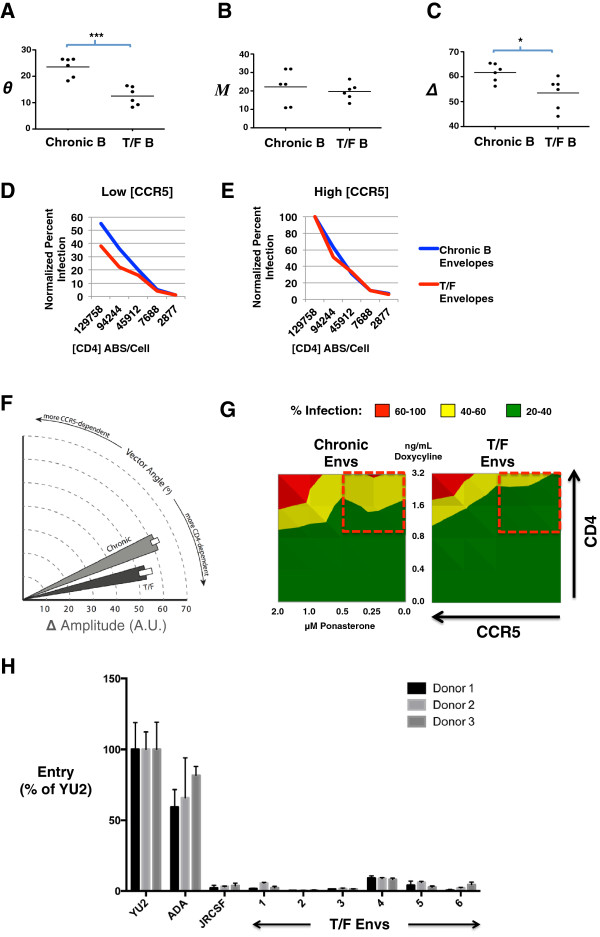
**Sensitivity vector metrics reveal differences in CD4/CCR5 usage efficiencies between Transmitter/Founder (T/F) and chronic envelopes.** Normalized infection data using T/F and chronic Env clones were analyzed using VERSA. **(A)** Vector angle, (*θ*), **(B)** mean infectivity (*M*), and **(C)** vector amplitude (Δ) values were obtained for each Env clone. Each Env was profiled twice, in triplicate, across 25 combinations of CD4/CCR5 expression. Average metrics of 6 individuals from each group (T/F or chronic, N=12) are shown, each group consisting of 900 data points. The median value of each metric for the T/F and chronic Env cohorts is marked by a line. p values were generated by the non- parametric unpaired *t* test (***p = 0.0003; *p = 0.05). **(D** and **E)** The normalized infectivity for the chronic (blue line) and T/F envelopes (red line) are averaged, and compared as a group at **(D)** low and **(E)** high levels of CCR5 expression, across varying levels of CD4 as indicated. **(F)** Wedge plot of the average angle and amplitude (+/- S.D.) obtained for T/F (dark grey) versus chronic envelopes (light grey). **(G)** The infectivity profile of individual T/F and chronic Envs (from Additional file 5: Figure S3) were averaged to form their respective group profile. 2-D contour plots representing the averaged infectivity profiles of T/F and chronic envelopes are shown. **(H)** T/F Envs and macrophage tropic (YU2, ADA) and non-macrophage tropic (JRCSF) R5 Envs were used to produce Env pseudotyped luciferase reporter viruses, which were subsequently titrated on JC53 cells. Monocyte derived macrophages were inoculated with equivalent infectious units of each reporter virus, and luciferase activity measured in cell lysates at 72hrs post infection. Results of infection in 3 independent donors are shown. Results are means of triplicate wells, and error bars represent standard deviations.

An accumulating body of evidence indicates that the majority of primary infections are established by a single viral clone [47-49]. To discern whether relevant differences in entry efficiencies exist between T/F and chronic Envs, we used the GGR Affinofile system to examine the infectivity of T/F Envs (isolated from acutely infected Feinberg stage II or III patients) [50], and compared their Affinofile GGR metrics (*θ, ∆, M*) with those from a standard panel of chronic Envs. The specific clones used are indicated in [see Additional file 2: Table S1]. The infectivity of each T/F and chronic Env was examined at 25 distinct CD4/CCR5 expression levels [see Additional file 3: Figure S2A-B], and their infectivity metrics (Figure 5A-C) were obtained via VERSA as described in methods.

Figure 5A shows that T/F Envs have a median θ that is significantly lower than that of chronic Envs (15° vs 25°, p = 0.0003), and that this lower θ was associated with a lower ∆ (vector amplitude) (Figure 5C). This correlation indicates a diminished responsiveness (lower ∆) that is weighted toward CD4 (lower *θ*), meaning T/F Envs take advantage of increases in CD4 expression less efficiently than Chronic Envs. The decreased responsiveness to CD4 is most evident at lower, more physiological levels of CCR5 expression, illustrated in Figure 5D and E. The wedge plot in Figure 5F summarizes the distinct T/F and chronic Env phenotypic differences in and observed within the cohort of subtype B Envs examined. Finally, the 2-D contour plots of the averaged infectivity between T/F and chronic Envs across the spectrum of CD4/CCR5 expression levels corroborate the differences indicated by their infectivity metrics: that at low to moderate levels of CCR5 (0–0.5 μM Pon), even the highest level of CD4 allowed only moderate entry levels (40-60%) for the T/F Envs (Figure 5G, compare upper right quadrants). This phenotype is consistent with the observation that T/F Envs, despite being universally CCR5-using, are almost always primary T-cell tropic (high CD4/low CCR5) and not macrophage-tropic (low CD4/high CCR5) [30]. We confirmed that all six of these R5 T/F Envs are indeed non-macrophage-tropic (Figure 5H).

### Affinofile metrics reveal that HIV-1 Envs exhibit subtype-specific differences in CD4/CCR5 usage efficiencies

**Figure 6:**
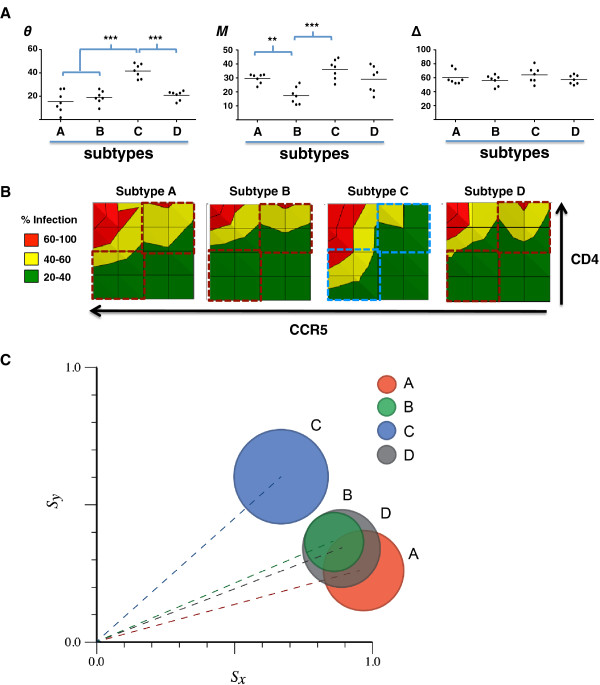
**HIV envelopes exhibit subtype-specific differences in CD4/CCR5 usage efficiencies. (A)** Normalized infection data from each subtype A, B, C and D envelope clones (n = 28) were analyzed by VERSA. The vector metrics were averaged for at least two independent infections (with a variance <5%) for each envelope in each subtype group. Vector angle (*θ*), mean infectivity (*M*), and vector amplitude (Δ) values for each envelope are shown as grouped by subtypes. P values were generated by the non- parametric unpaired *t* test (p*** < 0.005, **p < 0.05). **B)** 2-D contour plots of the average infectivity profile for each subtype, generated and color coded as in Figure 4G. The colored dashed square boxes compare the infectivity differences noted between subtype C (blue) Envs and others (red) in the lower left (LL) and upper right (UR) quadrants. Each Env clone was independently profiled twice. **(C)** Polar plot of the averaged sensitivity vectors obtained from each subtype, generated as in Figure 3E.

We next used the GGR Affinofile cells to characterize a panel of 28 subtype A, B, C and D Envs [see Additional file 4: Table S2]. As might be expected from a diverse panel of subtype Envs, there was a high degree of intra- and inter- subtype variability in all three metrics (Figure 6A). An additional figure shows the infectivity profile for each subtype Env examined [see Additional file 5: Figure S3]. Despite this variability, significant differences in CD4/CCR5 usage patterns between HIV-1 subtypes can be appreciated. For example, subtype C Envs had the highest θ and *M* values (Figure 6A), indicating that this subtype, as a group, used CCR5 more efficiently than Envs from other HIV-1 subtypes. The aggregate infectivity data confirms that subtype C Envs do, indeed, achieve a higher level of infection in response to increasing CCR5 levels, especially when CD4 levels are limiting (Figure 6B, compare the lower left quadrants). Interestingly, when CCR5 levels are low, subtype C Envs exhibited markedly reduced levels of infectivity compared to Envs from other HIV-1 subtypes, even at the highest CD4 levels (Figure 6B, compare upper right quadrants). Although this subtle nuance is not captured in ∆, infectivity profiles serve as an alternative method that adds depth to the existing algorithm. Finally, Envs from both HIV-1 subtypes A and C have significantly higher *M* values than subtype B Envs (Figure 6A). The polar plot in Figure 6C shows that subtype C envelopes can be clearly distinguished from other subtype envelopes based on their and metrics even if the amplitudes (∆) do not differ significantly between the subtypes.

### Affinofile profiling reveals that resistance to broadly neutralizing antibodies also results in reduced entry efficiency

Recent technological advancements have resulted in the cloning and characterization of numerous broadly neutralizing antibodies (BNabs) with increased potency and breath of coverage compared to the “classical” BNAbs such as b12, 2G12 and 2F5. PG9/PG16 and VRC01 represent two of the major classes of these “next generation” BNabs with non-overlapping epitopes [51-53]. Despite the breath and potency of these BNAbs, single point mutations, N160K and N279/280A, can confer resistance to PG9/PG16 and VRC01, respectively [51,53]. N160 and N279/280 are highly conserved residues across HIV-1 subtypes [See Additional file 6: Figure S4A], which suggest that these residues are under selective pressure.

**Figure 7:**
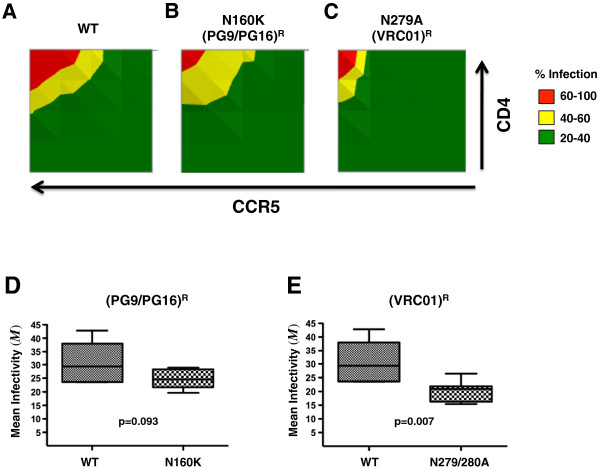
**Affinofile profiling reveals that resistance to broadly neutralizing antibodies (BNAbs) also results in reduced entry efficiency. (A-C)** N160K and N279/280A mutations were engineered into a random sample of 12 subtype A-D Envs. The resultant (PG9/PG16)^R^ and (VRC01)^R^ resistant Envs were assayed for CD4 and CCR5 usage efficiency along with their parental BNAb sensitive Envs. GGR Affinofile profiling was performed as previously described. 2-D contour plots of the averaged infectivity profiles for **(A)** WT, **(B)** (PG9/PG16)^R^, and **(C)** (VRC01)^R^ Envs are shown. The infectivity profile for the individual Envs are shown in supplementary Figure S5. Axes and color-codes are identical to previous contour plots. **(D-E)** The median values and interquartile ranges of the Mean infectivity (*M*) are shown for (PG9/PG16)^R^ or (VRC01)^R^ resistant Envs compared to their WT counterparts. P values calculated via a non-parametric paired *t*-test.

To determine potential entry efficiency consequences related to these BNab resistance mutations we generated resistant N160K and N279/280A mutants in 24 Envs representing subtypes A through D, and examined their CD4/CCR5 entry efficiencies using the GGR Affinofile system. Figure 7A, B and C, shows the mean infectivity profiles for wt Envs (n = 12, 3 each from subtype A-D), and their respective isogenic N160K, and N279/280A mutants, each Env examined across 25 distinct CD4/CCR5 expression levels. An additional figure shows the individual infectivity profile for all 36 Envs examined [see Additional file 6: Figure S4]. The PG9/PG16 (N160K) and VRC01 (N279/280A) resistance mutations reduce the efficiency of entry; both requiring higher levels of CD4 and CCR5 to achieve similar levels of infection as their wt counterparts. This can be appreciated by comparing the CD4/CCR5 expression level combinations that give rise to low levels of infection (green areas), or conversely, those that give rise to the highest level of infection (red areas), between the wt and mutant Envs (Figure 7A-C). This reduced entry efficiency phenotype across all subtypes tested is quantitatively reflected in the values, where the average *M* for PG9/PG16 and VRC01 resistant mutants is lower than that of their wt counterparts (Figure 7D and E). However, due to marked variability when comparing across all HIV-1 subtypes, only the difference between VRC01 resistance mutants and wt reached significance (p = 0.007). Our results suggest that resistance to BNAbs comes at the cost of reduced HIV-1 entry efficiency, and provides one functional explanation for the high conservation of these residues across HIV-1 subtypes. Both these reasons bode well for vaccine design that will elicit these kinds of BNAbs.

## Discussion

The Affinofle system and associated VERSA metrics have provided investigators a more quantitative method to characterize viral entry efficiency as a function of CD4 and CCR5 expression. Quantitative comparisons of these three VERSA metrics—Mean infectivity (*M*), Vector Angle (*θ*) and Amplitude (Δ)—have facilitated our understanding of how CD4/CCR5 usage efficiencies correspond to distinct Env phenotypes associated with resistance to CCR5-inhibitors, and the myriad of *in vitro* or *in vivo* selective pressures that result in differential or altered cell tropism [28,34,36,37,39,54-56].

### Efficiency of CD4/CCR5 usage and T cell subset tropism

A critical feature of our GGR Affinofile system is the ability to distill the aggregate entry phenotype of Env into three metrics. Here, we demonstrate that these VERSA metrics reflect biologically relevant phenotypes for wt JR-CSF Env, and two point mutants (S142N and K421D) known to modulate its affinity for CCR5, and the complex interplay of CD4 and CCR5 usage associated with mutations that affect conformational transitions (E153G). Specifically, S142N, which had larger *θ* and *M* values relative to wt JR-CSF, also infected total PMBCs better. This increase infectivity may be due to an expanded CD4+ T-cell subset tropism as S142N pseudotyped virions infected a larger portion of naïve T-cells relative to their wild-type counterparts (Figure 4C, 25.9% vs 6.8%). Intriguingly, naïve T-cells have undetectable levels of CCR5 by FACS [47,57,58], much like the CCR5 “FACS-negative” T-cell lines (Molt 4 and SupT1) that the S142N Env virus is known to infect in a CCR5-dependent manner [25]. Conversely, K421D, which had the smallest *θ* and *M* values, also infected PBMCs with the least efficiency, and lacked the expanded tropism seen with the S142N mutant.

Our GGR/Affinofile system can interrogate an Env phenotype across a fuller fitness landscape than traditional assays. The ability to evaluate infectivity across a broad spectrum of CD4 and CCR5 expression levels underscores the innate inter-dependence of CD4 and CCR5 levels in the context of infection. For example, although the enhanced macrophage tropism of the E153G mutant was originally attributed to increased CD4 binding affinity and more efficient infection on cells expressing low levels of CD4, our Affinofile assay describes an Env that is more responsive to changes in CCR5 than wt JR-CSF (*θ* = 38.3° and 30.9° respectively, Figure 3E). Our results complement and expand published results on E153G, and provide direct support for the proposed effect of E153G on V1/V2 loop flexibility, which can affect exposure of both the CD4 and CCR5 binding sites. The latest structural evidence also supports such a model [59].

What is the utility of being able to quantify the efficiency of CD4/CCR5 usage through a set of standardized metrics? For S142N, the ability to use lower levels of CD4 and CCR5 for entry correlates with its expanded tropism for naïve CD4+ T cells. HIV-1 preferentially infects memory, rather than naïve CD4+ T cells [60-62]. However, loss of naïve T-cells is also clearly associated with immune system decline and disease progression, but is thought to be due to secondary factors such as lymph node fibrosis, which destroys the regenerative niche required for maintaining naïve T-cells [63,64]. To our knowledge, the infection of naïve T-cells in lymph nodes of late stage patients have not been directly examined. Since late stage R5 isolates are also more efficient at using low levels of CD4 and CCR5 for entry [12,65], it is possible that infection of naïve T-cells by late stage R5 Envs might contribute to the diminishment seen. Currently, macrophage-tropism is widely used as a surrogate measure for R5 Envs that can use low levels of CD4 and/or CCR5 for entry [66], but it is not clear whether macrophage-tropic Envs also have an expanded tropism for naïve CD4+ T-cells. Use of our GGR Affinofile system and VERSA metrics to characterize extended panels of R5 macrophage-tropic and R5 non-macrophage tropic Envs will help shed light on this important issue related to R5 Env pathogenesis. Intriguingly, even a binary read-out, such as an increased ability to infect CD4^low^/CCR5^high^ relative to CD4^high^/CCR5^high^ Affinofile cells, has been observed in CSF-derived R5 Envs from a patient many months *before* the patient developed HIV-1 associated dementia [67]. Thus, it would also be of interest to determine the VERSA metrics of R5 Envs from a broader array of longitudinal cohorts to evaluate whether a certain pattern of VERSA metrics is predictive of pathogenicity or disease progression.

### T/F and chronic Envs

~70-80% of heterosexual or IV drug use HIV-1 transmission cases are established by a single transmitted/founder (T/F) virus clone [50,68-71]. Concerted efforts have been made to discern genotypic and phenotypic differences between T/F and chronic Envs, since such differences may inform vaccine design, shed light on the biology of HIV-1 transmission and pathogenesis, or facilitate development of strategies to prevent HIV-1 transmission [47,48]. While T/F Envs are enriched in genotypic features such as an overall reduction in the number of potential N-linked glycosylation sites (PNGS) [72], no unique genetic signatures can be ascribed only to T/F Envs. Phenotypic differences between T/F and chronic Envs also appear subtle: no overt differences were found in multiple assays such as entry/fusion efficiency into cells expressing high or low levels of CD4/CCR5, infection of CD4+ T-cell subsets, dendritic cell mediated *trans*-infection, and sensitivity to entry inhibitors [73-77]. However, moderate increases in sensitivity to neutralization by the CD4 binding site antibody b12, and more marked resistance to sCD4 inhibition, have been reported for some cohorts of subtype B T/F Envs relative to chronic Envs [75,76].

Our Affinofile profiling of a small panel of subtype B T/F and chronic Envs reveals moderate but significant differences in the CD4/CCR5 usage efficiencies. The differences are subtle, but the combination of *θ* and Δ clearly distinguishes the T/F Envs from the chronic Envs (Figure 5F). These data also indicate that T/F Envs are less efficient at using CD4, as a diminished responsiveness (lower ∆) is associated with CD4 (lower *θ*) without a significant change in overall infectivity (*M*) (Figures 5A, B and C). The implied decrease in CD4 usage efficiency exhibited by the T/F Envs in our study is consistent with the aforementioned cohort of T/F Envs with increased resistance to sCD4 neutralization [76]. However, sensitivities to sCD4 or b12 neutralization are surrogate markers for CD4 utilization, and neither directly measures the true entry phenotype of a virus with regards to CD4/CCR5 usage efficiency. sCD4 sensitivity does not always correlate with gp120-CD4 binding affinity ( [78] and references therein), and b12 neutralization can be affected by epitope changes that don’t affect CD4 binding [79]. For example, T/F Envs are enriched for the loss of a particular N-glycan site, mediated by *not* having a Thr at position 415 (T415X), that allows better access to key b12 binding residues at positions 417–419 [72]. Thus, the increased sensitivity to b12 neutralization may be associated with a genetic signature (T415X) enriched in T/F Envs, rather than being a general property of T/F Envs *per se*. In our cohort, there is no obvious relationship with sensitivity to b12 or sCD4 neutralization even though all but one T/F Env has the T415X signature [see Additional file 2: Table S1]. Yet, infectivity profiling across the full spectrum of CD4/CCR5 expression levels and VERSA metrics were able to reveal differences in entry phenotypes between T/F and chronic Envs. Clearly, our findings need to be extended by examination of larger groups. However, recent evidence suggests that T/F envs and chronic Envs can differ in their ability to use the maraviroc bound form of CCR5, but this phenotype is more obviously revealed only on CD4^high^/CCR5^high^ Affinofile cells [80]. The ability to use the MVC-bound form of CCR5 in this case is likely a surrogate marker for an expanded promiscuity in the use of CCR5 conformations. These results are consistent with our current findings and suggest that the full Affinofile profiling may have the requisite sensitivity to reveal subtle but real differences in Env phenotypes related to HIV-1 transmission.

The pattern of responses to CD4 and CCR5 observed using the Affinofile system and their correlation to infection on primary cells with different CD4 and CCR5 expression levels are naturally sensitive to alternate CCR5 conformations and post-translational modifications [81-89] that may or may not support entry. To achieve the most representative measure of CCR5 in the context of HIV entry, expression is quantified in terms of cell-surface epitopes specifically recognized by the broadly and potently neutralizing CCR5 Mab 2D7, a biologically relevant, surrogate measure of the majority of CCR5 that is accessible and functional as bona fide entry coreceptors [90]. However, we cannot rule out that some Envs can use qualitatively different conformations of CCR5 that are not represented by 2D7 antibody binding sites.

### Subtype Env specific differences

Subtype C viruses, in pure or recombinant forms, comprise the majority of HIV-1 infections worldwide, and are associated with heterosexual transmission. Subtype Envs do exhibit phenotypic differences as evidenced by a significant correlation between CCR5 and FPRL1 usage for subtype A and C Envs, and between CCR5 and CCR3 usage only for subtype B Envs [91,92]. These differences in alternate coreceptor usage in highly permissive NP2/CD4/CoR cells likely reflects the different evolutionary histories of the subtype Envs, and is more apt to be a surrogate marker for the efficiency of CCR5 usage or the use of a specific conformation of CCR5.

Subtype C Envs are indeed transmitted more efficiently *in utero* than subtype A or D Envs [93]. Thus, it seems reasonable to intuit that subtype C Envs are more efficient in cell entry and/or transmission. However, in vitro and ex vivo assays indicate that viruses bearing subtype C Envs are invariably outcompeted by other subtype Envs in PBMC outgrowth assays [94-96]. This decrease in replicative fitness presents an explanatory conundrum that may be illuminated by our Affinofile data. Our GGR Affinofile profiling results indicate that the average subtype C Env used CCR5 more efficiently than the other subtype Envs, but this was only true at low to moderate levels of CD4 (Figure 6B, compare lower left quadrants). Future refinements of the metric algorithm can provide more detail to these subtle nuances. At high levels of CD4 but lower (more physiologic) levels of CCR5 such as would be present on activated PBMCs (Figure 6B, compare upper right quadrants), subtype C Envs are *less* efficient at entry. The difference in entry efficiencies between subtype C and the other subtype Envs, reflected in the UR and LL quadrants of their infectivity profile (Figure 6B), might provide an explanatory framework that accounts for both the decreased replicative fitness observed in vitro (on activated PBMCs), and the notion that subtype C Envs must be more efficient at entry and/or transmission at some level. The VERSA metrics and infectivity profiles in Figure 6 quantify a genuine phenotypic difference between subtype C and other subtype Envs, and can serve as a reference point for future studies into their physiological correlates. Despite the small number of Envs examined (n = 28, 7 for each subtype), these are well-characterized reference subtype Envs, chosen carefully to represent acute/early infection isolates, so as to compare the Env phenotypes that might be specific to each subtype before disease stage-specific selective pressures come into play [see Additional file 4: Table S2].

### BNAb resistance mutations

Our Affinofile profiling suggests that mutations in Env that confer resistance to at least two BNAbs come at a fitness cost. This is perhaps not surprising since the mutated residues N160 and N279/280 are themselves highly conserved amongst HIV-1 subtypes suggesting that selective pressures are at play. Nevertheless, we engineered mutations into 12 Envs from 4 different subtypes, and observed a general trend that N279/280A (VRC01)^R^ mutations, and to a lesser extent, the N160K (PG9/PG16)^R^ mutations decrease the mean infectivity without a significant impact on the other two VERSA metrics. While the (VRC01)^R^ mutation near the CD4bs was likely to affect entry efficiency, it was not clear that the (PG9/PG16)^R^ mutation would. Indeed, the impact on entry efficiency is much greater for the (VRC01)^R^ mutation compared to the (PG9/PG16)^R^. It remains to be seen if resistant mutations to the latest generation of BNAbs all come at a fitness cost or whether they are epitope dependent. We recognize that our results regarding the impact of BNAb resistant mutations on entry efficiency need to be confirmed and expanded with a larger set of mutants and antibodies. Our GGR Affinofile system provides an appropriately high throughput methodology to facilitate such future studies. The results from these further studies might inform the engineering of the most appropriate immunogen that will elicit the BNAbs that will best constraint the development of resistance.

## Conclusions

In sum, Affinofile profiling not only interrogates the functional plasticity of HIV-1 Env in response to a spectrum of CD4 and CCR5 expression levels, it provides and distills the multi-dimensional data that captures this functional plasticity. Thus, Affinofile profiling may be a more sensitive method for discerning subtle but real differences in entry phenotypes that are not detected by other standard assays for evaluating CD4/CCR5 usage efficiency. A database of carefully curated VERSA metrics will help standardize the phenotypic characteristics of Envs from multiple cohorts and facilitate future studies into pathophysiology associated with Env phenotypes. We are currently creating a panel of GGR Affinofile cell lines that express alternate coreceptors as well as hybrid and mutant CCR5 that will help extend and refine such studies.

## Methods

### Virus production

Envelopes and the SG3∆env vector were obtained through the NIH AIDS and Research and Reference Reagent Program. Details and provenance of all envelopes used are given in Additional file 2: Table S1 and Additional file 4: Table S2. Pseudovirons were generated by cotransfection of 293T cells with Env-deleted SG3∆env vector and Env expressing vector at a 3:1 ratio with Bioline Bio T transfection reagent. 72 hours post transfection, viral supernatant was collected, clarified by low speed centrifugation and stored at -80**°**C. The number of infectious virus particles was determined by titration on Ghost HI-R5 cells, as described previously [97].

### CD4 and CCR5 cell surface expression

CD4 and CCR5 surface expression levels were determined by quantitative flow cytometry (qFACS) as described previously [34,39].

### GGR vector cloning

pNL-GFP-RRE was obtained through the NIH AIDS Research and Reference Reagent Program [40,41]. pNL-GFP-RRE was digested with SacI and SalI. The *Gaussia* luciferase gene was PCR amplified from pCMV-Gluc (Promega). The PCR product was digested with SacI and SalI and subsequently ligated into the precut pNL-GFP-RRE vector to form pNL-GGR.

### GGR virus production

GGR-expressing lentiviral transducing viruses were produced by cotransfection of 293T cells with pNL-GGR vector, pCMV∆R8.2, and pVSV-G at a ratio of 10:10:1, respectively, using the calcium phosphate method. Two days post transfection the viral supernatant was collected, clarified by low speed centrifugation, and filtered through a .45μM filter. Viral supernatant was then concentrated by ultracentrifugation at 32,000 x g for 90 minutes and stored at -80C.

### GGR single cell cloning

Affinofile cells were seeded into a 48 well plate at 5 X 10^4^ per well. 24 hours later cells were infected with 1 μg (p24 equivalents) of VSV-G pseudotyped GGR virus. Infected cells were then spinoculated for 2 hours at 37 degrees and 770 x g. Cells were washed once with PBS and replenished with fresh D10/B media. Cells were allowed to grow in a 10cm culture dish for three weeks, by splitting and replenishing media every 2–3 days. Single cell clones were then obtained by limiting dilution into 96-well plates. Single cell clones were passaged for three weeks, and clones with stable integration of the pNL-GGR vector were screened for optimal signal to noise ratio of *Gaussia* luciferase activity in the supernatant upon infection with JR-CSF virus. Selected clones were then screened for ones that still maintained a robust CD4 and CCR5 inducible response to doxycycline and ponasterone A.

### T cell infection

Leukopacks from healthy uninfected donors were obtained from the virology core at the UCLA CFAR. For purification of CD4+ T-cells, buffy coats containing peripheral blood mononuclear cells (PBMC) were first Ficoll-purified, and CD8+ T cells were depleted using Invitrogen CD8 Dynabeads. CD8 depleted PBMCs were incubated in RPMI supplemented with IL-2, 20% FCS and stimulated with CD3/CD28 coupled Dynabeads (Invitrogen) for three days. Three days post-stimulation, cells were washed twice and infected with indicated virus. Infection was synchronized by spinoculation for 2 hours at 2,000 rpm (770x *g*) at 4°C. After spinoculation, infectious media was replaced with fresh media. Three days post infection cells were collected and stained for T-cell subset markers CD4 (RPT-4), CD3 (OKT3), CCR7 (3D12) CD45RA (Hl100) (Ebiosciences), and intracellular p24 (KC57, BD Pharmingen).

### GGR affinofile assay

GGR Affinofile cells were seeded in a 96 well plate at 2 X 10^4^ cells/well. Simultaneously, cell surface expression of CD4 and CCR5 was induced with 0 to 4.0 ng/mL of Doxycycline and/or 0 to 2 μM of Ponasterone A, respectively. 18hrs later the induction media was removed. Each well of cells was then inoculated with HIV-1 at an MOI of 0.25, as determined on Ghost R5 cells. The cells were then spinoculated (770 x *g*) for 2 hours at 37° C. Infectious supernatant was then replaced with fresh D10 media (DMEM with 10% FBS and 1% Pen/Strep). At the indicated timepoints (hours post-infection) used in the various assays, 10 μl of supernatant was combined with 10 μl of substrate detection buffer (SDB: 50mM Tris–HCl (pH 7.5), 20% glycerol, 0.1% TritonX-100, 10mM DTT). The supernatant and SDB mix was assayed for *Gaussia* luciferase (GLuc) activity using Coelenterazine substrate in 96-well black plates according to manufacturer’s instructions (NEB, Ipswich, MA). GLuc-catalyzed bioluminescence was detected on the TECAN Infinite® M1000 microplate reader via luminescence scanning with an integration time of 8 seconds. All test were done with mararviroc controls to confirm exclusive CCR5 coreceptor usage.

### Data analysis

The Affinofile infectivity metrics were derived from raw or normalized data using the VERSA (Viral Entry Receptor Sensitivity Analysis) computational platform as previously described [34]. The considerations for the use of raw versus normalized data, and the limitations of each have been extensively reviewed [39].

## Competing interests

The authors declare they have no competing interests.

## Authors’ contributions

KC generated the system, performed the major experiments, and drafted the manuscript. NEW helped with the analysis, wrote some of the programs for data presentation, and finalized the figures and text for submission. TC formulated the mathematical analysis and VERSA metrics, and helped in interpreting the data. KB and JS performed the confirmatory assays on primary cells. PRG participated at all stages of the manuscript, providing invaluable input during multiple revisions. BL conceived of the study, and participated in its design and coordination and helped to draft the manuscript. All authors read and approved the final manuscript.

## Supplementary Material

Additional file 1: Figure S1 Isolates with different CD4 and CCR5 usage can be represented by distinct 3-D surface plots. GGR Affinofile cells induced to express 25 different combinations of CD4 and CC5 were infected with the (A) “CD4-independent” R5 SIV316, (B) R5X4 89.6, or (C) X4 IIIB pseudotyped viruses. The SIV 316 infection profile indicated that SIV 316 is much more sensitive to changes in CCR5 levels, and is relatively insensitive to varying CD4 levels. Conversely, the HIV IIIB infectivity profile indicated a phenotype that was dependent on changes in CD4, but was relatively insensitive to changes in CCR5. This phenotype can be attributed to the use of low levels of CXCR4 present on the HEK293 cells, the parental derivative of GGR Affinofile cells. The 89.6 virus demonstrated an infectivity profile that was equally sensitive to changes in CD4 and CCR5 levels. The distinct infectivity profiles for each Env demonstrated in A-C can be mathematically transformed into the corresponding 3-D surface plots shown in D-F. These three envelopes represent the diverse range of infectivity profiles that can be demonstrated in GGR Affinofile cells. (G) A polar plot representing the three metrics describing the infectivity profiles of the three viruses is shown. SIV316 has a vector angle closest to 90 degrees indicating a greater infective response to CCR5 expression and reflecting the CD4-independence of this Env. Conversely, HIV IIIB has a vector angle closest to zero degrees, endorsing an X4 tropism that is manifested as CCR5 independence. 89.6 has a vector angle of ~45 degrees indicating that it is equally sensitive to changes in CD4 and CCR5 levels. Each circle represents one independent experiment profiling infectivity across 25 distinct CD4/CCR5 expression levels. Click here for file

Additional file 2: Table S1 List of T/F and chronic envelopes. Click here for file

Additional file 3: Figure S2 Infectivity profiles of Chronic and T/F Envelopes. The infectivity profile for individual chronic (A) and T/F (B) derived envelopes across a spectrum of CD4 and CCR5 expression levels were generated and plotted as described in the Materials and Methods. One representative experiment out of two is shown. Each infectivity data point was performed in triplicate. The contour plots are arranged from highest to lowest mean infectivity (*M*), from left to right. (C) T/F Envs and macrophage tropic (YU2, ADA) and non-macrophage tropic (JRCSF) R5 Envs were used to produce Env pseudotyped luciferase reporter viruses, which were subsequently titrated on JC53 cells. Monocyte derived macrophages were inoculated with equivalent infectious units of each reporter virus, and luciferase activity measured in cell lysates at 72hrs post infection. Results of infection in 3 independent donors are shown. Results are means of triplicate wells, and error bars represent standard deviations. Click here for file

Additional file 4: Table S2 List of subtype envelopes. Click here for file

Additional file 5: Figure S3 Infectivity profiles of Subtype A-D Envelopes. The infectivity profile for indivudal Subtype A, Subtype B, Subtype C and Subtype D derived Envs (A-D, respectively) across a spectrum of CD4 and CCR5 expression levels were generated and plotted as described in the Materials and Methods. One representative experiment out of at least two is shown. The contour plots are arranged from highest to lowest mean infectivity (*M*), from left to right. Click here for file

Additional file 6: Figure S4 Infectivity profiles of (PG9/PG16)^R^ or (VRC01)^R^ Envs. (A) Consensus and/or predicted ancestral Env sequences from subtypes A-D were obtained from the Los Alamos HIV sequence database (http://www.hiv.lanl.gov), and the amino acid sequences from the relevant regions aligned. Arrows highlight location of conserved residues where single point mutations were engineered to confer PG9/16 (N160K) or VRC01 (N279/280A) resistance. (B-D) 2-D contour plots of the infectivity profile for individual Envs are shown for the wild-type parental WT (A), and the corresponding N160K (B), and N279/280A (C) mutants. Subtype specific Envs (A1-3, B1-3, C1-3) refer to the Env clones listed in Additional file 4: Table S2. Axes and color-codes are identical to previous contour plots. Contour plots are ordered based on the *M* values of the parent Env (highest to lowest, from left to right). Click here for file
