# Supplementary figures and images for: Distinct HIV-1 entry phenotypes are associated with transmission, subtype specificity, and resistance to broadly neutralizing antibodies
Source: Retrovirology. 2014 Jun 23;11:48. doi: 10.1186/1742-4690-11-48 (PMC4230403; doi:10.1186/1742-4690-11-48)

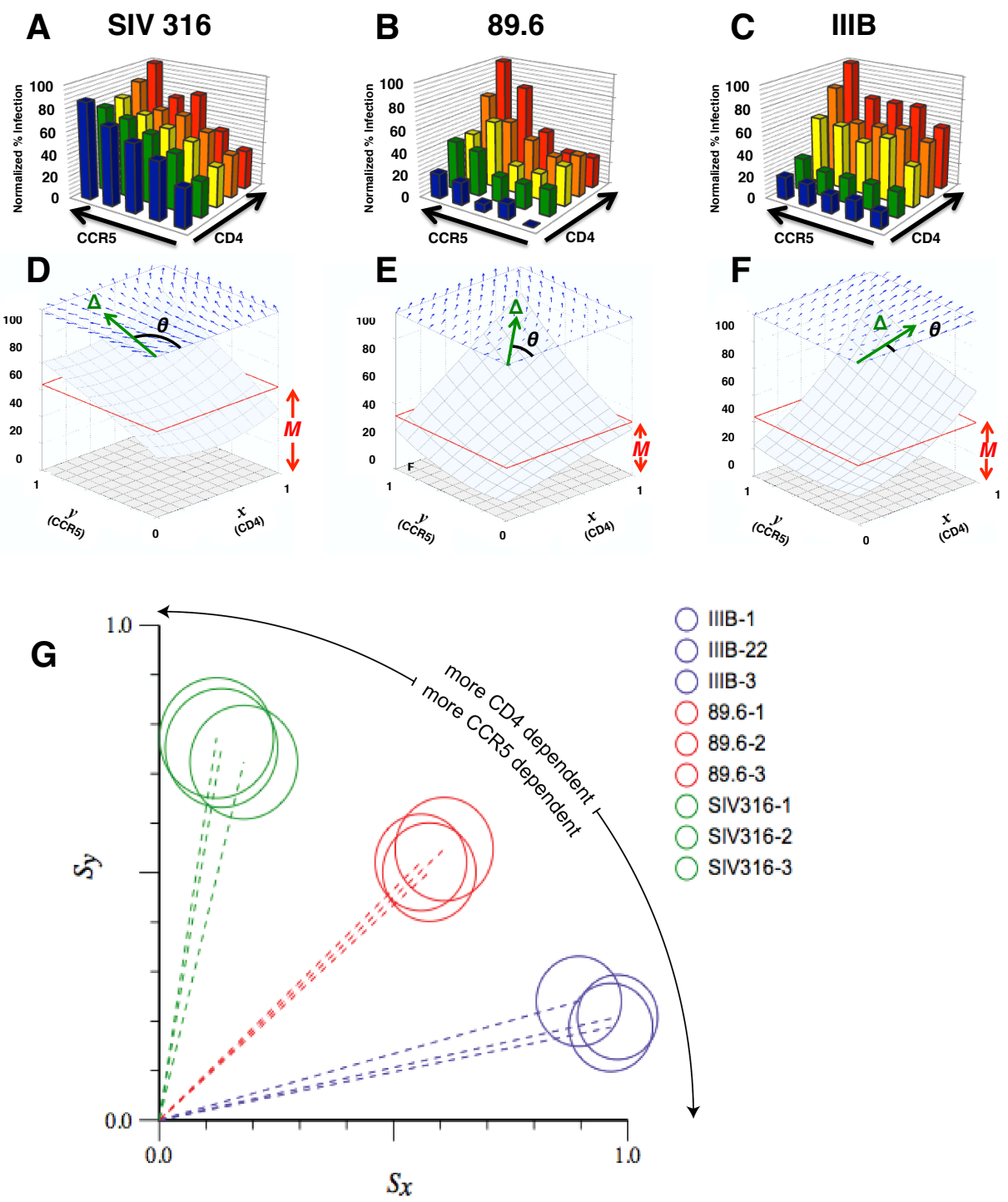

Supplement: Additional file 1: Figure S1 — Isolates with different CD4 and CCR5 usage can be represented by distinct 3-D surface plots. GGR Affinofile cells induced to express 25 different combinations of CD4 and CC5 were infected with the (A) “CD4-independent” R5 SIV316, (B) R5X4 89.6, or (C) X4 IIIB pseudotyped viruses. The SIV 316 infection profile indicated that SIV 316 is much more sensitive to changes in CCR5 levels, and is relatively insensitive to varying CD4 levels. Conversely, the HIV IIIB infectivity profile indicated a phenotype that was dependent on changes in CD4, but was relatively insensitive to changes in CCR5. This phenotype can be attributed to the use of low levels of CXCR4 present on the HEK293 cells, the parental derivative of GGR Affinofile cells. The 89.6 virus demonstrated an infectivity profile that was equally sensitive to changes in CD4 and CCR5 levels. The distinct infectivity profiles for each Env demonstrated in A-C can be mathematically transformed into the corresponding 3-D surface plots shown in D-F. These three envelopes represent the diverse range of infectivity profiles that can be demonstrated in GGR Affinofile cells. (G) A polar plot representing the three metrics describing the infectivity profiles of the three viruses is shown. SIV316 has a vector angle closest to 90 degrees indicating a greater infective response to CCR5 expression and reflecting the CD4-independence of this Env. Conversely, HIV IIIB has a vector angle closest to zero degrees, endorsing an X4 tropism that is manifested as CCR5 independence. 89.6 has a vector angle of ~45 degrees indicating that it is equally sensitive to changes in CD4 and CCR5 levels. Each circle represents one independent experiment profiling infectivity across 25 distinct CD4/CCR5 expression levels. [file 1742-4690-11-48-S1.pdf]

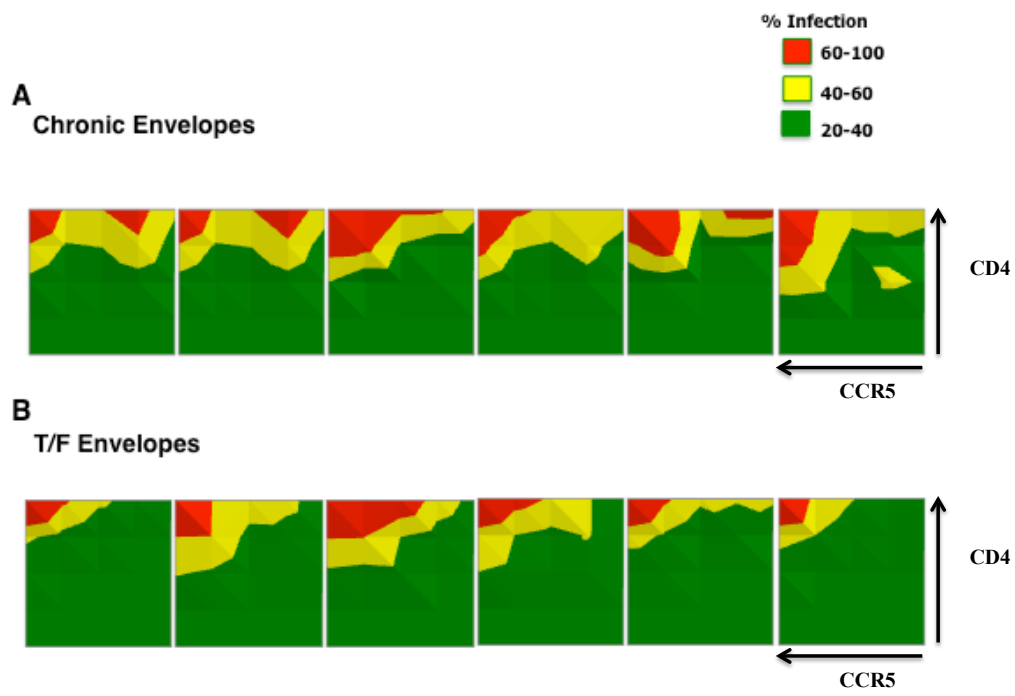

Supplement: Additional file 3: Figure S2 — Infectivity profiles of Chronic and T/F Envelopes. The infectivity profile for individual chronic (A) and T/F (B) derived envelopes across a spectrum of CD4 and CCR5 expression levels were generated and plotted as described in the Materials and Methods. One representative experiment out of two is shown. Each infectivity data point was performed in triplicate. The contour plots are arranged from highest to lowest mean infectivity (M), from left to right. (C) T/F Envs and macrophage tropic (YU2, ADA) and non-macrophage tropic (JRCSF) R5 Envs were used to produce Env pseudotyped luciferase reporter viruses, which were subsequently titrated on JC53 cells. Monocyte derived macrophages were inoculated with equivalent infectious units of each reporter virus, and luciferase activity measured in cell lysates at 72hrs post infection. Results of infection in 3 independent donors are shown. Results are means of triplicate wells, and error bars represent standard deviations. [file 1742-4690-11-48-S3.pdf]

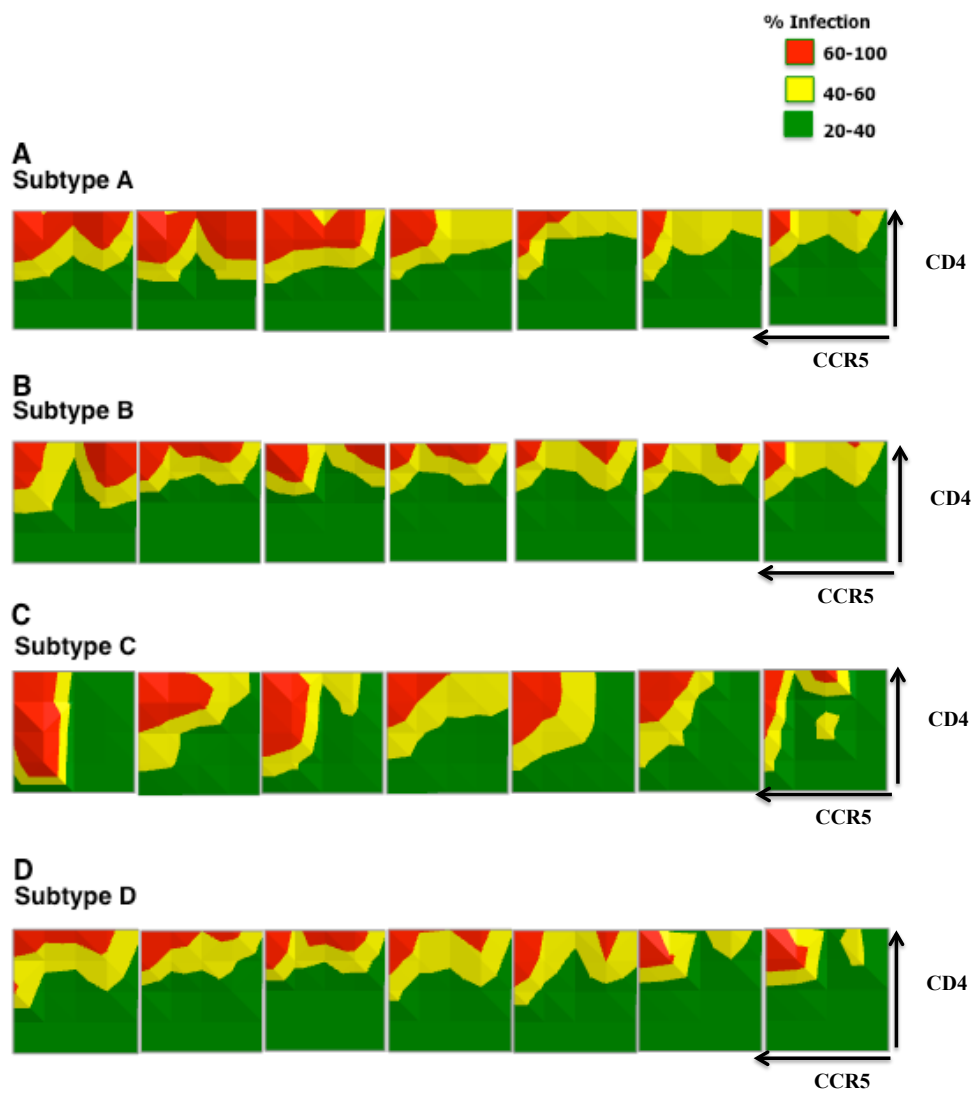

Supplement: Additional file 5: Figure S3 — Infectivity profiles of Subtype A-D Envelopes. The infectivity profile for indivudal Subtype A, Subtype B, Subtype C and Subtype D derived Envs (A-D, respectively) across a spectrum of CD4 and CCR5 expression levels were generated and plotted as described in the Materials and Methods. One representative experiment out of at least two is shown. The contour plots are arranged from highest to lowest mean infectivity (M), from left to right. [file 1742-4690-11-48-S5.pdf]
