# Supplementary material for: Distinct HIV-1 entry phenotypes are associated with transmission, subtype specificity, and resistance to broadly neutralizing antibodies
Source: Retrovirology. 2014 Jun 23;11:48. doi: 10.1186/1742-4690-11-48 (PMC4230403; doi:10.1186/1742-4690-11-48)
Supplement: Additional file 2: Table S1 — List of T/F and chronic envelopes. [file 1742-4690-11-48-S2.pdf]

Additonal file 2. Supplementary Table 1. List of T/F and Chronic Envelopes

Env type	env clone	Gender	Age	Feinberg Stage	Viral Load (copies/ml)	Disease Status	Location	Risk factor	Accession Number	AIDS Repository Designation	b12 IC_{50} (ug/ml)	sCD4 IC_{50} (nM)	T415 (Yes/No)
T/F	p6244_13.B5.4576	M		II	274,000	NA	USA	SPD	EU289191	p6244_13.B5.4576	>50	254	N
	p63358.p3.4013	NR		II	260,000	NA	USA	SPD	EU289192	p63358.p3.4013	>50	538	N
	p700010040.C9.4520	F		II	741,499	NA	USA	IVDU	EU289193	p700010040.C9.4520	0.7	97	N
	p1054.TC4.1499	M		II	320,000	NA	USA	SPD	EU289185	p1054.TC4.1499	4.2	113	Y
	pPRB926_04.A9.4237	NR		II	756,000	NA	USA	SPD	EU289197	pPRB926_04.A9.4237	0.5	93	N
	pSC45.4B5.2631	M		II	6,318,529	NA	Trinidad	Heterosexual	EU289201	pSC45.4B5.2631	0.7	268	N

Env type	env clone	Gender	Age	Time since sero-conversion	CD4 Count (cells/mm^3)	Disease Status	Location	Risk factor	Accession Number	AIDS Repository Designation
Chronic	92TH014.12	M	38	25.6	ND	AS	Bangkok, Thailand	IVDU	U08801	pSVIII-92TH014.12
	92US711.14	M	44	17	853	AS	Baltimore, USA	IVDU	U08448	pBA301711.14
	92US712.4	F	35	15	537	AS	Baltimore, USA	IVDU	U08449	pBA301712.4
	92US714.1	M	28	12	546	AS	Baltimore, USA	IVDU	U08450	pBA301714.1
	92US715.6	M	36	20	470	AS	Baltimore, USA	IVDU	U08451	pBA301715.6
	92US716.6	M	39	4	787	AS	Baltimore, USA	IVDU	U08452	pBA301716.6

NA, non-applicable
AS, asymptomatic
NR, not recorded
**Risk behavior where known. Subjects listed as "SPD" were source plasma donors who denied having sex for money, homosexual activity, IVDU, or receiving a blood transfusion or a tattoo in the preceding year.
