# Supplementary material for: Distinct HIV-1 entry phenotypes are associated with transmission, subtype specificity, and resistance to broadly neutralizing antibodies
Source: Retrovirology. 2014 Jun 23;11:48. doi: 10.1186/1742-4690-11-48 (PMC4230403; doi:10.1186/1742-4690-11-48)
Supplement: Additional file 4: Table S2 — List of subtype envelopes. [file 1742-4690-11-48-S4.pdf]

# Additional File 4. Supplemental Table 2. List of Subtype Envelopes

| Env type | Env clone | Approximate length of time of infection | Viral Load (copies/ml) | CD4 Count (cells/mm$^3$) | Location | Mode of Transmission | Accession Number | Reference | Group PI | b12 $IC_{50}$ (ug/ml) | sCD4 $IC_{50}$ (mg/ml) |
|---|---|---|---|---|---|---|---|---|---|---|---|
| A1 | Q259env.w6 | 81 dpi | 2,000,000 | NA | Kenya | NA | AF407151 | 1 | Overbaugh | >20 | NA |
| A2 | QB726.70M.ENV.B3 | 70 dpi | 61,940 | NA | Kenya | NA | FJ866111 | 1 | Overbaugh | >20 | NA |
| A3 | QH359.21M.ENV.C1 | 21 dpi | 32,120 | NA | Kenya | NA | FJ866121 | 2 | Overbaugh | >20 | NA |
| A4 | QH209.14M.ENV.A2 | 14 dpi | 28,600 | NA | Kenya | NA | FJ866118 | 2 | Overbaugh | >20 | NA |
| A5 | QF495.23M.ENV.A3 | 23 dpi | 217,050 | NA | Kenya | NA | FJ866114 | 2 | Overbaugh | >20 | NA |
| A6 | Q769env.h5 | 61 dpi | 9,000,000 | NA | Kenya | NA | AF407159 | 2 | Overbaugh | >20 | NA |
| A7 | QH343.21M.ENV.A10 | 21 dpi | 40,750,000 | NA | Kenya | NA | FJ866119 | 2 | Overbaugh | >20 | NA |
| | | | | | | | | | | | |
| B1 | SC 422661.8 (SVPB8) | 4 wks | 1,380,000 | ND | Trinidad | F-M | A Y835441 | 3 | Montefiori | 4.7 | 0.2 |
| B2 | pCAAN5342 clone A2 | NA | >1,000,000 | 278 | USA | M-M | A Y835452 | 3 | Montefiori | >50 | 16 |
| B3 | pREJO4541 clone 67 | 2 wks | 722,349 | 848 | USA | F-M | A Y835449 | 3 | Montefiori | 0.7 | 0.5 |
| B4 | pTRJO4551clone 58 | 1 wks | 8122951 | NA | USA | M-M | A Y835450 | 3 | Montefiori | >50 | 20.2 |
| B5 | AC10.0, clone 29 | 4 wks | 40,700 | 919 | USA | M-M | A Y835446 | 3 | Montefiori | 1.8 | 8.5 |
| B6 | QH0692, clone 42 | 6 wks | 9,611 | NA | Trinidad | F-M | A Y835439 | 3 | Montefiori | 0.3 | 0.5 |
| B7 | pRHPA4259 clone 7 | <8 wks | 1,458,354 | 247 | USA | M-F | A Y835447 | 3 | Montefiori | 0.1 | 1.8 |
| | | | | | | | | | | | |
| C1 | HIV-16055-2, | 2 dpi | 534,557 | 830 | India | F-M | EF117268 | 4 | Montefiori | >50 | 11.4 |
| C2 | HIV-16845-2, | 20 dpi | 199,655 | 579 | India | M-F | EF117269 | 4 | Montefiori | >50 | 1 |
| C3 | HIV-25710-2, | 19 dpi | 3523 | 350 | India | F-M | EF117271 | 4 | Montefiori | >50 | 2.6 |
| C4 | HIV-25711-2, | 4 dpi | 6,633,880 | 471 | India | F-M | EF117272 | 4 | Montefiori | 25.9 | 29 |
| C5 | HIV-26191-2, | 9 dpi | 5,346,070 | 338 | India | F-M | EF117274 | 4 | Montefiori | 4.9 | 17.1 |
| C6 | HIV-00836-2, | 85 dpi | 31,104 | ND | India | M-F | EF117265 | 4 | Montefiori | >50 | >50 |
| C7 | HIV-001428-2, clone 42 | 11 dpi | 217,812 | 454 | India | M-F | EF117266 | 4 | Montefiori | >50 | 5.2 |
| | | | | | | | | | | | |
| D1 | QA013.70I.ENV.H1 | 70 dpi | 1,527,700 | NA | Kenya | NA | FJ866134 | 2 | Overbaugh | >20 | NA |
| D2 | QA013.70I.ENV.M12 | 70 dpi | 1,527,700 | NA | Kenya | NA | FJ866135 | 2 | Overbaugh | >20 | NA |
| D3 | QA465.59M.ENV.D1 | 59 dpi | 37,750 | NA | Kenya | NA | FJ866137 | 2 | Overbaugh | 17.16 | NA |
| D4 | QD435.100M.ENV.B5 | 100 dpi | 17,470 | NA | Kenya | NA | FJ866140 | 2 | Overbaugh | >20 | NA |
| D5 | QD435.100M.ENV.A4 | 100 dpi | 17,470 | NA | Kenya | NA | FJ866139 | 2 | Overbaugh | >20 | NA |
| D6 | QD435.100M.ENV.E1 | 100 dpi | 17,470 | NA | Kenya | NA | FJ866141 | 2 | Overbaugh | >20 | NA |
| D7 | QA465.59M.ENV.A1 | 59 dpi | 37,750 | NA | Kenya | NA | FJ866136 | 2 | Overbaugh | 9.09 | NA |
| | | | | | | | | | | | |

*may be defined differently in different studies; please specify
time since last seronegative test, times since acue retroviral conversion syndrome, combination of clinical parameters

NA, Not available

| Reference | | Group PI |
|---|---|---|
| EM Long et al, ARHR 2002 | **1** | Julie Overbaugh |
| CA Blish et al, JV 2009 | **2** | Julie Overbaugh |
| M Li et al, JV 2005 | **3** | David Montefiori |
| SS Kulkarni et al, Virology 2009 | **4** | David Montefiori |
