# Supplementary material for: Distinct HIV-1 entry phenotypes are associated with transmission, subtype specificity, and resistance to broadly neutralizing antibodies
Source: Retrovirology. 2014 Jun 23;11:48. doi: 10.1186/1742-4690-11-48 (PMC4230403; doi:10.1186/1742-4690-11-48)
Supplement: Additional file 6: Figure S4 — Infectivity profiles of (PG9/PG16)R or (VRC01)R Envs. (A) Consensus and/or predicted ancestral Env sequences from subtypes A-D were obtained from the Los Alamos HIV sequence database (http://www.hiv.lanl.gov), and the amino acid sequences from the relevant regions aligned. Arrows highlight location of conserved residues where single point mutations were engineered to confer PG9/16 (N160K) or VRC01 (N279/280A) resistance. (B-D) 2-D contour plots of the infectivity profile for individual Envs are shown for the wild-type parental WT (A), and the corresponding N160K (B), and N279/280A (C) mutants. Subtype specific Envs (A1-3, B1-3, C1-3) refer to the Env clones listed in Additional file 4: Table S2. Axes and color-codes are identical to previous contour plots. Contour plots are ordered based on the M values of the parent Env (highest to lowest, from left to right). [file 1742-4690-11-48-S6.pdf]

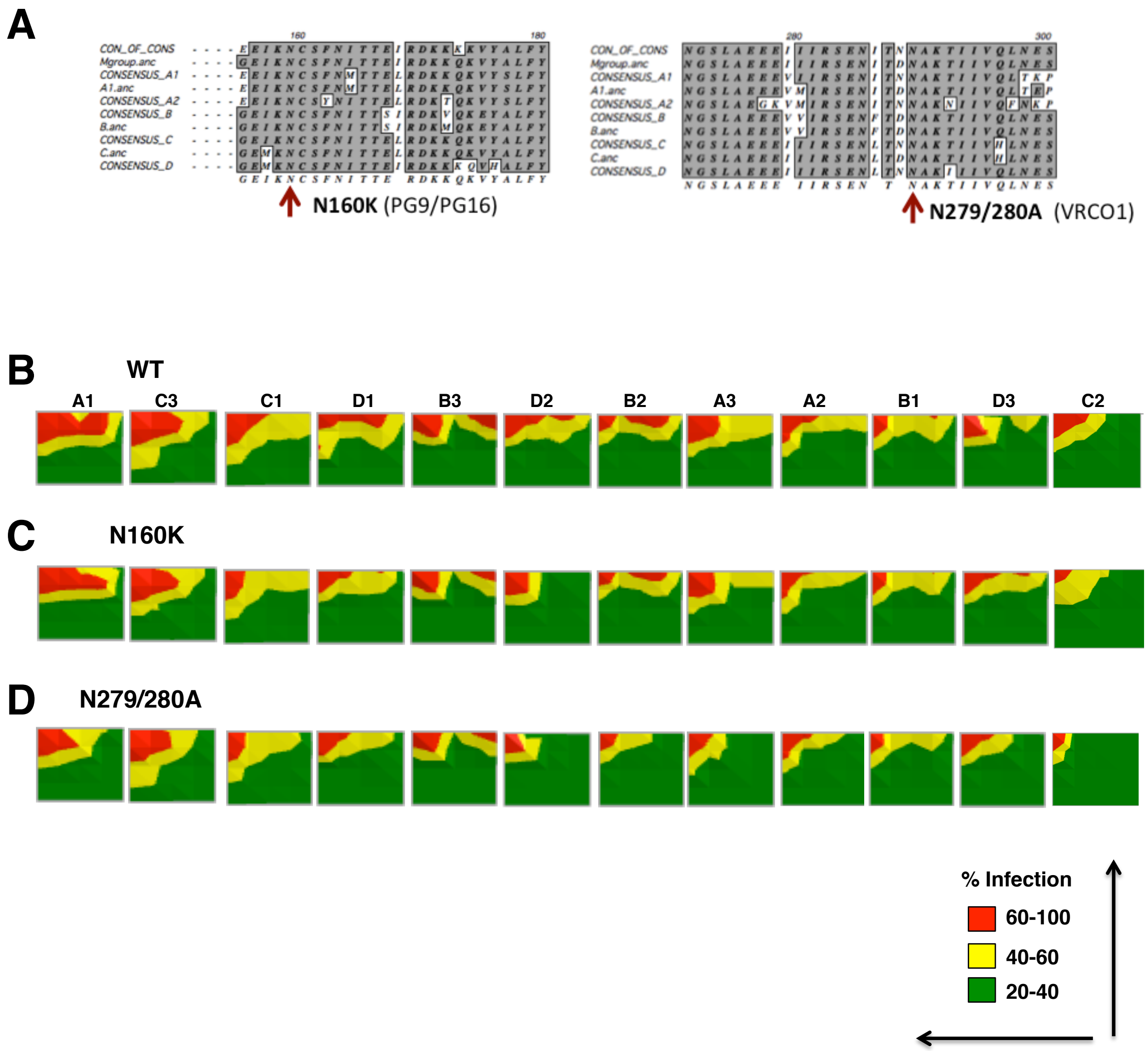

Additional File 6.
